# Inadequate Performance of ChatGPT on Orthopedic Board-Style Written Exams

**DOI:** 10.7759/cureus.62643

**Published:** 2024-06-18

**Authors:** Chandler A Sparks, Matthew J Kraeutler, Grace A Chester, Edward V Contrada, Eric Zhu, Sydney M Fasulo, Anthony J Scillia

**Affiliations:** 1 Department of Orthopedic Surgery, Hackensack Meridian School of Medicine, Nutley, USA; 2 Department of Orthopedics, University of Colorado Anschutz Medical Campus, Aurora, USA; 3 Department of Orthopedic Surgery, St. Joseph’s Medical Center, Paterson, USA; 4 Department of Sports Medicine/Orthopedics, Seton Hall University, Paterson, USA

**Keywords:** artificial intelligence, orthopedic education, chatgpt, chatgpt-3.5, orthopedic surgery, orthopedic exam

## Abstract

Background: Chat Generative Pre-Trained Transformer (ChatGPT) is an artificial intelligence (AI) chatbot capable of delivering human-like responses to a seemingly infinite number of inquiries. For the technology to perform certain healthcare-related tasks or act as a study aid, the technology should have up-to-date knowledge and the ability to reason through medical information. The purpose of this study was to assess the orthopedic knowledge and reasoning ability of ChatGPT by querying it with orthopedic board-style questions.

Methodology: We queried ChatGPT (GPT-3.5) with a total of 472 questions from the Orthobullets dataset (*n* = 239), the 2022 Orthopaedic In-Training Examination (OITE) (*n* = 124), and the 2021 OITE (*n* = 109). The importance, difficulty, and category were recorded for questions from the Orthobullets question bank. Responses were assessed for answer choice correctness if the explanation given matched that of the dataset, answer integrity, and reason for incorrectness.

Results: ChatGPT correctly answered 55.9% (264/472) of questions and, of those answered correctly, gave an explanation that matched that of the dataset for 92.8% (245/264) of the questions. The chatbot used information internal to the question in all responses (100%) and used information external to the question (98.3%) as well as logical reasoning (96.4%) in most responses. There was no significant difference in the proportion of questions answered correctly between the datasets (*P* = 0.62). There was no significant difference in the proportion of questions answered correctly by question category (*P* = 0.67), importance (*P* = 0.95), or difficulty (*P* = 0.87) within the Orthobullets dataset questions. ChatGPT mostly got questions incorrect due to information error (i.e., failure to identify the information required to answer the question) (81.7% of incorrect responses).

Conclusions: ChatGPT performs below a threshold likely to pass the American Board of Orthopedic Surgery (ABOS) Part I written exam. The chatbot’s performance on the 2022 and 2021 OITEs was between the average performance of an intern and to second-year resident. A major limitation of the current model is the failure to identify the information required to correctly answer the questions.

## Introduction

Chat Generative Pre-Trained Transformer (ChatGPT) is an advanced artificial intelligence (AI) chatbot that uses deep learning algorithms trained on vast written resources, including the internet, books, and other text, to generate human-like responses for a seemingly infinite number of inquiries [1]. Since its release in November 2022, the AI chatbot has gained significant public interest and has generated discussion regarding its role in society, including education, customer service, literature, and research, as a few examples [2]. Its use for healthcare tasks, however, requires that the AI chatbot can reason through medical information to prevent errors, biases, spreading of misinformation, or even harm to patients [3]. Such tasks include personalized patient interaction, consumer health education, acting as a personalized patient, and acting as a study aid, to name a few [4].

ChatGPT has displayed some ability to reason through medical information on the United States Medical Licensing Exam (USMLE), performing marginally better than the passing threshold, which supports its application to assist learners in medical education [4,5]. However, previous studies have found that ChatGPT (GPT-3.5) is unlikely to reach a passing threshold on the orthopedic board examination [3,6]. These studies also assessed ChatGPT’s performance by question taxonomy as well as ChatGPT’s ability to find verifiable sources.

The purpose of this study was to further investigate ChatGPT’s performance across different orthopedic surgery education resources and question types as well as to provide further analysis of the output contents. Such analysis may provide a better understanding of the AI model’s strengths as well as limitations and areas for future improvement. Specifically, we quantified ChatGPT’s performance (GPT-3.5) across two different resources widely used for orthopedic surgery resident education (Orthopedic In-Training Examination [OITE] and Orthobullets). We also evaluated the correctness of ChatGPT’s justification as well as performance across question categories, importance, and difficulty. Finally, our analysis assessed the rationale for incorrect outputs and response integrity, specifically evaluating the logical justification employed and the use of intrinsic and extrinsic information in outputs.

## Materials and methods

Datasets and question selection

Three datasets were used to assess ChatGPT’s (GPT-3.5) ability to reason through multiple-choice, orthopedic board-style questions. A total of 239 questions were randomly selected from the Orthobullets (Lineage Medical) [7] free question bank, a widely used question bank among orthopedic surgery residents, by generating an exam where all 3,747 questions had the potential to be included. All questions that met inclusion criteria from the two most recent (2022 and 2021) OITEs, a test that is administered to United States orthopedic surgery residents each year to assess clinical knowledge, were also used. Questions that used any images were excluded given that ChatGPT (GPT-3.5) is not capable of directly reading images. Questions that used tables were also excluded due to the limited ability to place tables with correct formatting into the ChatGPT text input field. Questions were copied and pasted into the text input field exactly as they appeared in the original dataset, with multiple-choice answers separated into a new line for each answer. ChatGPT was queried with questions from the Orthobullets dataset during May 2023 and with questions from the OITEs during June 2023. The question category, importance, and difficulty were recorded for questions from the Orthobullets dataset but not the OITEs, as this information is not given by the OITE dataset. All queries were made in separate chats to ensure that ChatGPT would not draw information from inquiries previously made in the same chat.

Output analysis

Output analysis methods were adapted from Gilson et al. [4] and used for all datasets. To summarize, the multiple-choice answer selected by ChatGPT was first compared to the correct multiple-choice answer per the dataset. If correct, the justification given by ChatGPT was then compared to the justification given by the dataset. If ChatGPT did not attempt logical justification, we asked it to give a justification by asking it, “Why did you select this answer?” and then comparing the justification given to that given by the dataset. If incorrect, it was recorded if ChatGPT directly stated that it was guessing or might be wrong, if it did not directly state this but recommended follow-up with an additional resource or expert (e.g., physician), or if it did not state either of these.

Qualitative analysis was performed by trained medical students (CAS, EVC, GAC, EZ) using the dataset explanations to guide analysis and working in collaboration to reconcile all uncertain labels. All ChatGPT responses were qualified using the following three binary variables: Logical reasoning, Internal information, and External information. An output from the chatbot was recorded as having Logical reasoning if it analyzed and evaluated options and made deductions as well as logical inferences based on available information, even if incorrect. An output was recorded as having Internal information if it used knowledge, data, or facts that were already present within the input (e.g., within the question itself). An output was recorded as having External information if it used knowledge, data, or facts that were not present in the input (i.e., not present within the question).

For all outputs where ChatGPT chose the incorrect answer choice, the reason for the incorrect answer choice was classified as one of the following four errors: logical error, information error, statistical error, or multiple errors. Incorrect output is classified as a logical error if the pertinent information was identified but the chatbot failed to convert the information into the correct answer choice (e.g., correctly identifies that a patient’s presentation is consistent with chronic prosthetic joint infection but incorrectly states the next best step in management for an acute prosthetic joint infection). Output was recorded as being incorrect due to Information error if it incorrectly answered a question due to failure to identify information either internal to the question or from an external source that would be expected knowledge (e.g., incorrectly stating that the ideal candidate for submuscular bridge plating is *an adult with a femur fracture*). Output was recorded as being incorrect due to Statistical error if an incorrect answer choice was chosen due to an arithmetic mistake, either explicit (e.g., failure to correctly execute calculation) or indirect (e.g., failure to estimate prevalence). An incorrect answer choice was recorded as being due to Multiple errors if more than one of the previously described errors contributed to an incorrect answer choice by ChatGPT.

Statistical analysis

All statistical analysis was performed in RStudio (Version 1.4.1106; Posit PBC). A chi-squared test of independence was used to compare the number of questions ChatGPT correctly and incorrectly answered among the Orthobullets dataset vs. 2022 OITE vs. 2021 OITE. Comparisons were also made between the questions correctly and incorrectly answered by question category, importance, and difficulty from the Orthobullets dataset.

## Results

ChatGPT performance

**Figure 1:**
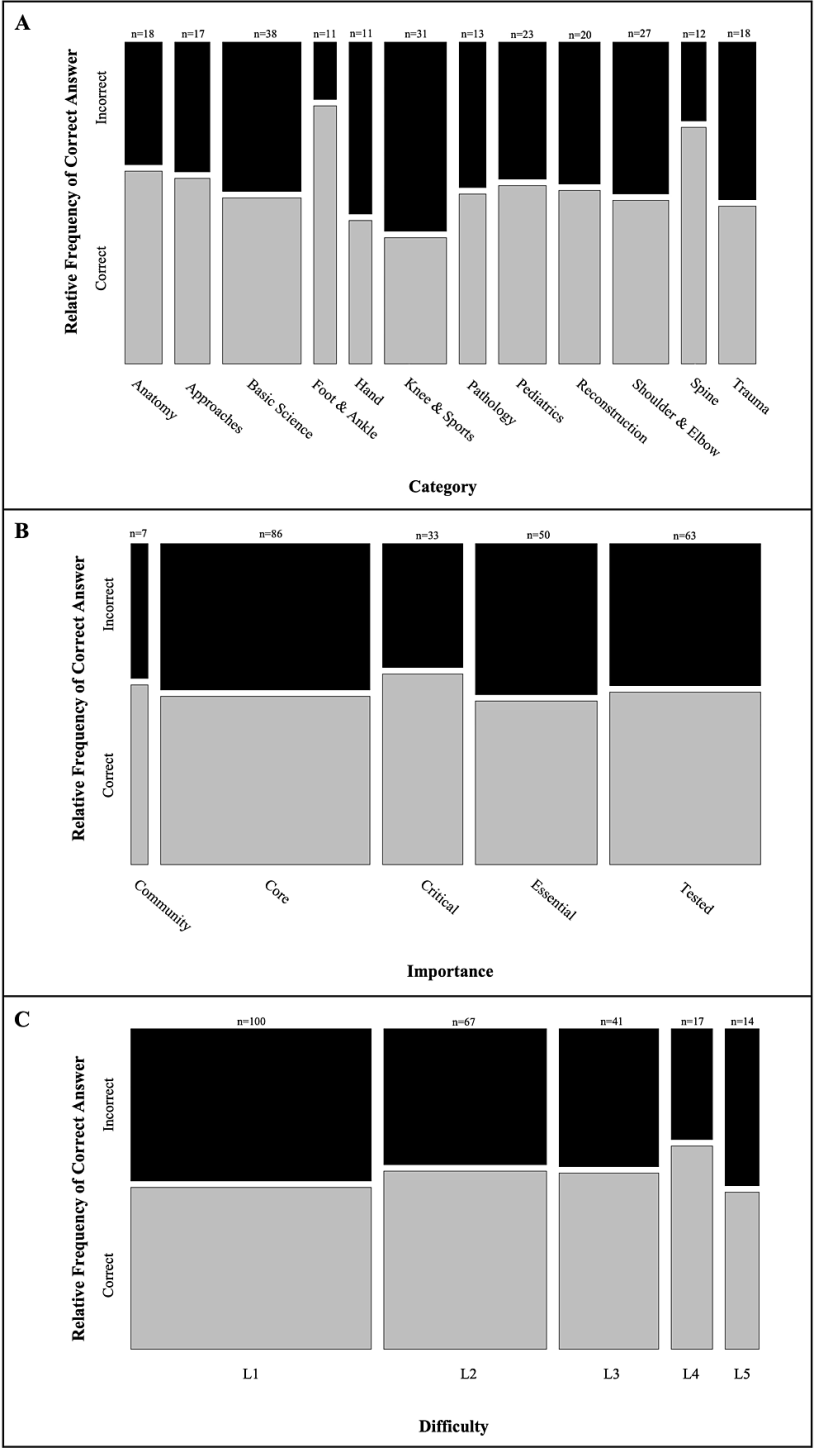
ChatGPT correct vs. incorrect answers by question characteristics. Figures represent the relative frequency of ChatGPT correct (gray) vs. incorrect (black) responses by (A) question category, (B) importance, and (C) difficulty. Column height by color represents the relative frequency of correct vs. incorrect answers and column width represents the relative sample size by category. All data were collected from the Orthobullets dataset (*n* = 239).

Altogether, 472 questions were included in this study. A total of 124 questions met the criteria from the 2022 OITE and 109 from the 2021 OITE. A total of 239 questions were included from the Orthobullets dataset. Overall, ChatGPT correctly answered 55.9% (264/472) of the questions. There was no significant difference in the proportion of questions answered correctly between the Orthobullets dataset, 2022 OITE, and 2021 OITE (*P* = 0.62; *χ*^2^ = 0.97; degrees of freedom [df] = 2). These results are summarized in Table 1. Out of the questions answered correctly, ChatGPT provided a justification that matched the dataset in 92.8% (245/264) of the outputs. Among the 239 questions from the Orthobullets dataset, there was no significant difference in the proportion of questions answered correctly by question category (*P* = 0.67; *χ*^2^ = 8.45; df = 11), importance (*P* = 0.95; *χ*^2^ = 0.70; df = 4), or difficulty (*P* = 0.87; *χ*^2^ = 1.25; df = 4). These results are summarized in Figure 1.

**Table 1:** ChatGPT performance by dataset (Orthobullets vs. 2022 Orthopedic In-Training Exam [OITE] vs. 2021 OITE).

Source	Orthobullets (*n* = 239)	2022 OITE (*n* = 124)	2021 OITE (*n* = 109)	Total (*n* = 472)
Correct	131 (54.8%)	74 (59.7%)	59 (54.1%)	264 (55.9%)
Incorrect	108 (45.2%)	50 (40.3%)	50 (45.9%)	208 (44.1%)

Qualitative analysis of outputs

ChatGPT attempted logical justification in 96.4% (455/472) of the outputs. Information internal to the question was used in 100% of the outputs, and information external to the question in 98.3% (464/472) of the outputs. These results are summarized in Table 2.

**Table 2:** Frequency of logical information, internal information, and external information used by ChatGPT by dataset (Orthobullets vs. 2022 Orthopedic In-Training Exam [OITE] vs. 2021 OITE).

Source	Orthobullets (*n* = 239)	2022 OITE (*n* = 124)	2021 OITE (*n* = 109)	Total (*n* = 472)
Logical justification	225 (94.1%)	122 (98.4%)	108 (99.1%)	455 (96.4%)
Internal information	239 (100%)	124 (100%)	109 (100%)	472 (100%)
External information	233 (97.5%)	122 (98.4%)	109 (100%)	464 (98.3%)

Incorrect response analysis

ChatGPT most commonly answered questions incorrectly due to Information error (81.7%). Logical errors (9.6%), statistical errors (1.4%), or multiple errors (7.2%) were minor causes for ChatGPT to incorrectly answer a question. When multiple errors were made, these were information and logical (13/15, 86.7%) and information and statistical (2/15, 13.3%). These results are summarized in Table 3. 

**Table 3:** Reason for incorrect ChatGPT response by dataset (Orthobullets vs. 2022 Orthopedic In-Training Exam [OITE] vs. 2021 OITE).

Source	Orthobullets (*n* = 108)	2022 OITE (*n* = 50)	2021 OITE (*n* = 50)	Total (*n* = 208)
Information error	79 (73.2%)	44 (88.0%)	47 (94.0%)	170 (81.7%)
Logical error	15 (13.9%)	4 (8.0%)	1 (2.0%)	20 (9.6%)
Statistical error	1 (0.9%)	2 (4.0%)	0 (0%)	3 (1.4%)
Multiple errors	13 (12.0%)	0 (0%)	2 (4.0%)	15 (7.2%)

Of the 208 incorrect outputs, ChatGPT directly stated that it was guessing or might be incorrect in 6 (2.9%) of those outputs. It did not directly state that it was guessing or might be incorrect but did recommend follow-up with another resource or expert in 47 (22.6%) of the incorrect outputs. It did not state either of these in 155 (74.5%) of the incorrect outputs. 

## Discussion

The most important finding of this study is that ChatGPT’s overall performance was 55.9%, which is lower than the minimal passing correlate to the American Board of Orthopedic Surgery (ABOS) Part I written examination on the previous three OITEs [8-10]. The score corresponding to the ABOS Part I minimal passing standard was 69.2% and 68.8% correct on the 2021 and 2022 OITEs, respectively [9,10]. ChatGPT performed inferior to these standards at 54.1% (2021 OITE) and 59.7% (2022 OITE) correct. Therefore, the ChatGPT performed below the standard expected of senior orthopedic surgery residents. In 2021, the mean score on the OITE among residents at Accreditation Council for Graduate Medical Education (ACGME) programs was 51% and 60% among postgraduate year (PGY)-1 and PGY-2 residents, respectively [9]. In 2022, the mean score among PGY-1 and PGY-2 residents at ACGME programs was 55% and 61%, respectively [10]. Thus, ChatGPT’s (GPT-3.5) medical knowledge and ability to reason through medical information was closer to that of an intern or PGY-2 resident and below that of PGY-3 to PGY-5 residents. For the technology to be used as a tool for obtaining information about orthopedic surgery, it should have up-to-date knowledge and the ability to deliver accurate and complete responses to inquiries. Our analysis suggests that it is currently inadequate for these purposes but is encouraging for prospects with improvements.

This study did not find any question type, category, or difficulty level where ChatGPT showed decreased utility compared to others. It often gave thorough responses that applied information from other resources. However, it frequently failed to identify the proper information required to answer the questions, for example, the ideal candidates for a procedure, contraindications, current guidelines, etc., with this being its largest limiting factor to correctly answering questions. An explanation for this may be the way ChatGPT obtains its information. Specifically, ChatGPT draws its information from the vast resources it is trained on, which may contain contrasting or unreliable information that causes the chatbot difficulties in answering questions. One suggestion for such improvements is limiting the resources that the AI model is trained on to only peer-reviewed medical literature, similar to PubMedGPT 2.7B [6,11]. This improvement may overcome the challenge of identifying the proper information needed to accurately answer medical questions that we identified in the present study. Such an improvement may allow the chatbot to act as a resource for the retrieval of accurate and personalized responses to questions about specific orthopedic concepts, diagnoses, or treatments. Given its current limitations, however, the technology is currently inadequate for these purposes and may misinform orthopedic surgery trainees. 

Other studies have also found ChatGPT (GPT-3.5) to be unlikely to pass the ABOS Part I written examination and perform closer to a PGY-1 resident [3,6]. Our findings are in agreement with these studies but utilize questions from various sources and provide a more in-depth analysis of the factors that may be limiting the technology’s performance. Other studies evaluating ChatGPT’s performance on specialty-specific exams have found ChatGPT (GPT-3.5) to perform lower than expected of residents to complete residency (plastic surgery and dermatology), while others have found it to exceed the passing threshold (neurosurgery) or nearly reach the passing threshold (radiology) [12-15]. It is not completely understood why ChatGPT’s performance on some standardized multiple-choice exams is substandard (e.g., orthopedic surgery) but not others. It is possible that ChatGPT is not trained on sources of information for orthopedic surgery that are as reliable as those for the other specialties, given that we found it to be largely limited in identifying the suitable information, though future investigation is needed to confirm this. 

There are limitations of this study to be noted. First, this study only assessed one of the currently available versions of the AI chatbot, though GPT-3.5 is still widely used due to its lower price point and faster speeds. Future studies will be needed to better understand the strengths and limitations of new models. Also, this study was limited to the two OITE exams and a series of randomly selected Orthobullets questions, and it is possible that ChatGPT’s performance would have been different on a different set of questions. However, we used a large sample size of questions and the two most recent OITE exams to avoid asking questions that are no longer relevant in orthopedics or whose correct answer could be argued based on recent evidence. Another study limitation is that ChatGPT’s knowledge cutoff is September 2021, and we included an OITE exam conducted in November 2022. However, the chatbot’s performance was worse on the 2021 OITE than the 2022 OITE. It is also worth mentioning that interrater reliability was not measured for labeling of the outputs. Finally, though the questions used in the present study are widely trusted to prepare orthopedic surgery residents for the ABOS Part I examination, an actual ABOS Part I written examination was not used in the present study, as these exams are not accessible.

## Conclusions

ChatGPT (GPT-3.5) performs below a threshold likely to pass the ABOS Part I written exam. The chatbot’s performance on the 2022 and 2021 OITEs was between the average performance of an intern to second-year resident. While the chatbot often gives thorough responses, it frequently fails to identify the correct information needed to correctly answer the questions. This may limit the utility of the chatbot for obtaining accurate orthopedic information. Further research with models trained on a limited number of trusted resources is warranted, given the limitations of the chatbot identified in this study.
